# Supplementary material for: Rate of force development in the quadriceps of individuals with severe knee osteoarthritis: A preliminary cross-sectional study
Source: PLoS One. 2022 Jan 11;17(1):e0262508. doi: 10.1371/journal.pone.0262508 (PMC8751984; doi:10.1371/journal.pone.0262508)
Supplement: S1 Table — (DOCX) [file pone.0262508.s001.docx]

**S1 Table.** Post hoc analysis to test the between-group differences adjusted for covariates in the quadriceps RFD for mild and severe KOA

|  | Mild KOA (n=58) | Severe KOA (n=8) | Between-group difference | 95% CI | Effect size | |
| --- | --- | --- | --- | --- | --- | --- |
|  |  |  |  |  | Hedges | 95% CI |
| Quadriceps RFD (%MVC/ms*kg) | 6.97±1.72 | 5.10±1.71 | **0.88** | **0.23–1.54** | **-1.07** | **-1.83 to -0.30** |

KOA: knee osteoarthritis; RFD: rate of force development; 95% CI: 95% confidence interval

Adjusted for age (years), sex, and knee pain VAS (mm)
