# Supplementary material for: Rate of force development in the quadriceps of individuals with severe knee osteoarthritis: A preliminary cross-sectional study
Source: PLoS One. 2022 Jan 11;17(1):e0262508. doi: 10.1371/journal.pone.0262508 (PMC8751984; doi:10.1371/journal.pone.0262508)
Supplement: S2 Table — (DOCX) [file pone.0262508.s002.docx]

**S2 Table.** Post hoc analysis to test the between-group differences adjusted for covariates in maximum quadriceps strength for mild and severe KOA

|  | Mild KOA (n=58) | Severe KOA (n=8) | Between-group difference | 95% CI | Effect size | |
| --- | --- | --- | --- | --- | --- | --- |
|  |  |  |  |  | Hedges | 95% CI |
| Maximum quadriceps strength (Nm/kg) | 1.49±0.41 | 1.39±0.69 | 0.06 | -0.09 to 0.21 | -0.22 | -0.96 to 0.52 |

KOA: knee osteoarthritis; 95% CI: 95% confidence interval

Adjusted for age (years), sex, and knee pain VAS (mm)
