# Supplementary material for: Rate of force development in the quadriceps of individuals with severe knee osteoarthritis: A preliminary cross-sectional study
Source: PLoS One. 2022 Jan 11;17(1):e0262508. doi: 10.1371/journal.pone.0262508 (PMC8751984; doi:10.1371/journal.pone.0262508)
Supplement: S3 Table — (DOCX) [file pone.0262508.s003.docx]

**S3 Table.** Differences between participants with mild and severe KOA in the quadriceps early (range 0–100 ms) RFD

|  | Mild KOA^*^ (n = 58) | Severe KOA^*^ (n=8) | p-value^†^ |
| --- | --- | --- | --- |
| Quadriceps RFD (%MVC/ms*kg) | 10.02±3.50 | 8.24±3.72 | 0.23 |

KOA: knee osteoarthritis; RFD: rate of force development

^*^ Values are expressed as mean ± SD or number (percentage)

^†^ Based on the unadjusted analysis (Mann–Whitney U-test) between participants with early and severe KOA
