# Supplementary material for: Rate of force development in the quadriceps of individuals with severe knee osteoarthritis: A preliminary cross-sectional study
Source: PLoS One. 2022 Jan 11;17(1):e0262508. doi: 10.1371/journal.pone.0262508 (PMC8751984; doi:10.1371/journal.pone.0262508)
Supplement: S4 Table — (DOCX) [file pone.0262508.s004.docx]

**S4 Table.** Differences between women participants with mild and severe KOA in the quadriceps RFD and maximum quadriceps strength

|  | Mild KOA^*^ (n = 50) | Severe KOA^*^ (n=6) | p-value^†^ |
| --- | --- | --- | --- |
| Quadriceps RFD (%MVC/ms*kg) | 7.14±1.76 | 5.09±1.99 | **0.02** |
| Maximum quadriceps strength (Nm/kg) | 1.42±0.36 | 1.30±0.70 | 0.50 |

KOA: knee osteoarthritis; RFD: rate of force development

^*^ Values are expressed as mean ± SD or number (percentage)

^†^ Based on the unadjusted analysis (Mann–Whitney U-test) between participants with early and severe KOA
