# Supplementary material for: Rate of force development in the quadriceps of individuals with severe knee osteoarthritis: A preliminary cross-sectional study
Source: PLoS One. 2022 Jan 11;17(1):e0262508. doi: 10.1371/journal.pone.0262508 (PMC8751984; doi:10.1371/journal.pone.0262508)
Supplement: Attachment — Submitted filename: Response to Reviewers.docx [file pone.0262508.s007.docx]

October 6, 2021

Dragan Mirkov, Ph.D.

Academic Editor

***PLOS ONE***

Manuscript ID: **PONE-D-21-21042**

**"Rate of force development in the quadriceps of individuals with severe knee osteoarthritis: a preliminary cross-sectional study"**

Dear Dr. Dragan Mirkov

We, the authors, thank you for your e-mail dated August 23, 2021, regarding the manuscript mentioned above. We appreciate the insightful comments of the editor and reviewer and have modified our manuscript in accordance with the comments. We hope that the changes made are appropriate. Revisions to the manuscript are shown in red font.

***Outline of Point-to-point Reply Letter***

Response to the journal requirements….………......…Author action #1 (Page 3-4)

Response to the comments of reviewer….………......…Author action #2-13 (Page 5−21)

**Responses to the journal requirements:**

**[Comment #1]**

We note that you have included the phrase “data not shown” in your manuscript. Unfortunately, this does not meet our data sharing requirements. PLOS does not permit references to inaccessible data. We require that authors provide all relevant data within the paper, Supporting Information files, or in an acceptable, public repository. Please add a citation to support this phrase or upload the data that corresponds with these findings to a stable repository (such as Figshare or Dryad) and provide and URLs, DOIs, or accession numbers that may be used to access these data. Or, if the data are not a core part of the research being presented in your study, we ask that you remove the phrase that refers to these data.

**[Author action #1]**

Thank you for your insightful and helpful suggestions. We have made some corrections in accordance with the comments from journal requirement, which hopefully meet the journal requirement. We hope that the changes made are appropriate. Revisions to the manuscript are shown in red font. The relevant part was corrected from "data not shown" to "Supplemental Table 3" (please see **Page 12/Lines 251–253 in the Discussion section**).

“Moreover, we did not observe a significant reduction in early (range 0–100 ms) RFD in severe KOA patients in this study (Supplemental Table 3).”

Moreover, the relevant results were added as supplemental data (please see **Page 21/Lines 422–425 in the Supplemental data section**).

**Table 3.** Differences between participants with mild and severe KOA in the quadriceps early (range 0–100 ms) RFD

|  | Mild KOA^*^ (n = 58) | Severe KOA^*^ (n=8) | p-value^†^ |
| --- | --- | --- | --- |
| Quadriceps RFD (%MVC/ms*kg) | 10.02±3.50 | 8.24±3.72 | 0.23 |

KOA: knee osteoarthritis; RFD: rate of force development

^*^ Values are expressed as mean ± SD or number (percentage)

^†^ Based on the unadjusted analysis (Mann–Whitney U-test) between participants with early and severe KOA

**Responses to the comments from the reviewer:**

**[Comment #2]**

RFD in different conditions (post-injury, post-surgery, KOA or hip OA related etc.) is always topic of high research and clinical interest. The authors should be commended for their work. The concerns I have are the same ones the authors mentioned in study limitations. One of major ones is the sample size, specifically, very small sample in the severe KOA group. I understand most of the time it is hard to increase the number of participants but since this is a cross-sectional study, I believe that is plausible. Nevertheless, the authors should be given a chance to provide substantial revisions of their work before being accepted for publishing.

**[Author action #2]**

Thank you for your insightful and helpful suggestions. We have made some corrections in accordance with the comments from reviewer, which hopefully meet the reviewer’s criteria. Revisions in the manuscript are shown in red font.

Thank you for this important reminder. As you pointed out, the small sample size of the severe KOA group is a major concern in this study. Therefore, in accordance with the reviewer's comments, we added the word "preliminary" to the title to emphasize that this is a preliminary study. Moreover, in the introduction, I emphasized why this study is important even though it is a preliminary study (please see **Page 4/Lines 86–89 in the Introduction section**).

“If the trend of the relationship between RFD and radiographic KOA can be clarified in a preliminary study with a small sample, it will be important information for early detection of severe KOA development and prevention of progression to severe KOA.”

Additionally, in the future, if the COVID-19 infection situation improves, we will consider increasing the sample size of the severe KOA group.

**[Comment #3]**

Title: consider adding word “preliminary” before cross-sectional study

**[Author action #3]**

Thank you for this important reminder. We have revised the following sentences (please see **Page 1/Lines 1–2 in the Title section**).

**“Rate of force development in the quadriceps of individuals with severe knee osteoarthritis: a preliminary cross-sectional study”**

**[Comment #4]**

Line 36. not decreased but lower, since this is a cross-sectional study

**[Author action #4]**

Thank you for this important reminder. We have revised the following sentences (please see **Page 2/Lines 34–37 in the Abstract section**).

“Sixty-six participants were analyzed. Individuals with severe KOA displayed significantly lower quadriceps RFD (p=0.009), the lower being greater than the lower in maximum quadriceps strength (between-group difference, ES: 0.88, -1.07 vs. 0.06, -0.22).”

**[Comment #5]**

Line 52: please elaborate modifiable risk factors in more details

**[Author action #5]**

Thank you for this important reminder. We have revised the following sentences (please see **Page 3/Lines 52–53 in the Introduction section**).

“Identifying and updating modifiable risk factors that can be improved by rehabilitation, such as muscle strength is, therefore, a critical unmet need.”

**[Comment #6]**

Line 68: it depends, some measures of RFD are largely independent from MVC torque thus represent another muscle function quality. For that reason, I here suggest using term “muscle function” rather than “muscle strength”

**[Author action #6]**

Thank you for this important reminder. We have revised the following sentences (please see **Page 3/Lines 67–69 in the Introduction section**).

“The rate of force development (RFD) is an index reflecting explosive muscle strength. The RFD has recently received attention as a measure of muscle function, distinct from maximum strength.”

**[Comment #7]**

Line 70: do you imply on maximum RFD? To my modest knowledge, that is a bit slow since maximum RFD is usually exerted well below 200 ms. Individuals recovering from ACL reconstructions reach maximum RFD at approximately 180 ms from the contraction onset. You need to specify that times stated are observed for older adults and those with KOA.

**[Author action #7]**

Thank you for this important reminder. This study does not focus on maximum RFD, but focus on early or late phase RFD close to activities of daily living. Therefore, we have revised the following sentences (please see **Page 3-4/Lines 69–72 in the Introduction section**).

“In terms of muscle contraction time, maximum strength requires more than 300 ms for exertion. Muscle activity in daily life is performed over a muscle contraction time until 50–200 ms [9, 10].”

**[Comment #8]**

Line 73: in facts, there are numerous papers published on that matter (check Mirkov et al. 2004)

**[Author action #8]**

Thank you for this important reminder. The study by Mirkov et al. 2004 was referenced and we have revised the following sentences (please see **Page 4/Lines 71–76 in the Introduction section**).

“Therefore, with regard to muscle contraction time, the RFD may be more closely related to daily activities than maximum strength. For example, previous study reported that the RFD was related to elbow movement performance [11]. Moreover, studies have reported that the RFD in KOA patients was significantly associated with the activities of daily living score [12] and biomechanical gait variables [13].”

**[Comment #9]**

Line 78: “in” instead of “of”

**[Author action #9]**

Thank you for this important reminder. We have revised the following sentences (please see **Page 4/Lines 77–79 in the Introduction section**).

“Moreover, patients with radiographically severe KOA (Kellgren and Lawrence [K&L] grade > 2) have significantly more impairments in daily activities than mild KOA patients (K&L grade 1-2) [14].”

**[Comment #10]**

Line 79: Consider beginning the sentence like “Thus, it is plausible that patients…”

**[Author action #10]**

Thank you for this important reminder. We have revised the following sentences (please see **Page 4/Lines 79–81 in the Introduction section**).

“Thus, it is plausible that patients with radiographically severe KOA may have a more significantly diminished quadriceps RFD than maximum quadriceps strength.”

**[Comment #11]**

Line 80: Consider using term “diminished” than “decreased” throughout the manuscript. It may be more suitable since this is cross-sectional study

**[Author action #11]**

Thank you for this important reminder. We have revised the following sentences (please see **Page 4/Lines 79–81 in the Introduction section**).

“Thus, it is plausible that patients with radiographically severe KOA may have a more significantly diminished quadriceps RFD than maximum quadriceps strength.”

**[Comment #12]**

Line 82: Please specify the aim(s) of the study.

**[Author action #12]**

Thank you for this important reminder. We have revised the following sentences (please see **Page 4/Lines 83–86 in the Introduction section**).

“The aim of the study is to test the hypothesis that: (1) individuals with severe KOA would have a significantly lower quadriceps RFD than individuals with early KOA and (2) the diminish in quadriceps RFD would be greater than the diminish in maximum quadriceps strength in individuals with severe KOA.”

**[Comment #13]**

Line 84: Please rewrite (see comment for Line 80)

**[Author action #13]**

Thank you for this important reminder. We have revised the following sentences (please see **Page 4/Lines 83–86 in the Introduction section**).

“The aim of the study is to test the hypothesis that: (1) individuals with severe KOA would have a significantly lower quadriceps RFD than individuals with early KOA and (2) the diminish in quadriceps RFD would be greater than the diminish in maximum quadriceps strength in individuals with severe KOA.”

**[Comment #14]**

Line 125: analysis instead of analyze

**[Author action #14]**

Thank you for this important reminder. We have revised the following sentences (please see **Page 6/Lines 130–132 in the Materials and Methods section**).

“To permit analysis the association of outcome measures with radiographic severity, the present sample was classified, as reported previously [17], into mild KOA (K&L grade 1–2) and severe KOA (K&L grade 3–4).”

**[Comment #15]**

Lines 141-142: According to this sentence one may understand that RFD and MVC were assessed separately, when in fact you used a single test (MVC performed as strong and as fast as possible) to assess both features of muscle function. Please rewrite.

**[Author action #15]**

Thank you for this important reminder. We have revised the following sentences (please see **Page 6-7/Lines 147–148 in the Materials and Methods section**).

“The results of quadriceps strength test were recorded in Newtons (N), and two repetitions were performed.”

**[Comment #16]**

Line 143: “Two repetitions were performed for each test” – again, it implies to something that wasn’t’ done

**[Author action #16]**

Thank you for this important reminder. We have revised the following sentences (please see **Page 6-7/Lines 147–148 in the Materials and Methods section**).

“The results of quadriceps strength test were recorded in Newtons (N), and two repetitions were performed.”

**[Comment #17]**

Line 151: Consider moving this sentence before the last one from previous paragraph.

**[Author action #17]**

Thank you for this important reminder. We have revised the following sentences (please see **Page 7/Lines 154–159 in the Materials and Methods section**).

“MVC in the quadriceps was defined as the maximal torque value obtained from two attempts. The MVC was normalized to body mass. We assessed the RFD over the first 200 ms of maximal isometric contraction, whereby the onset of contraction was deemed as the point at which torque had increased 4 Newton-meter (Nm) above the baseline value [20]. The RFD was analyzed at 200 ms from the onset of torque [21]. The RFD was normalized to body mass and MVC torque (%MVC) [20, 22]. The average of two repetitions was used for statistical analysis.”

**[Comment #18]**

Line 154: This sentence is redundant

**[Author action #18]**

Thank you for this important reminder. We have revised the following sentences (please see **Page 7/Lines 159–160 in the Materials and Methods section**).

“The RFD was normalized to body mass and MVC torque (%MVC) [20, 22].”

**[Comment #19]**

Line 155: Since you presented muscle strength as torque (Nm) why simply not presenting RFD data as RTD, and then continue with eliminating influence of body mass and MVC.

**[Author action #19]**

Thank you for this important reminder. The RFD data were originally corrected for body weight and %MVC values, but were revised to the following sentences (please see **Page 7/Lines 159–160 in the Materials and Methods section**) for better understanding.

“The RFD was normalized to body mass and MVC torque (%MVC) [20, 22].”

**[Comment #19]**

Line 157: Why reporting reliability data when this was among your hypotheses? Reliability of HHD has been show by others as good to excellent.

**[Author action #19]**

Thank you for this important reminder. The relevant sentence has been deleted from the manuscript.

**[Comment #20]**

Lines 177-179: it is not clear why the authors ran the ANCOVA. It is generally recommended that categorical variables as gender and age not to be used as covariates. For example, women constitute a majority of the sample studied. Why not simply remove all the males. I don’t think it would change your findings since you have only 2 males in the severe KOA group, but it would be much easier for future readers to comprehend the results and the findings. On the other hand, both MVC strength and RFD data were normalized with respect to body mass, and shin length. One more reason not to consider gender as covariate.

**[Author action #20]**

Thank you for this important reminder.

Removing males from the mild and severe KOA would reduce the overall sample size by 10 individuals. Since the sample size of this study was originally small, males were included in the analysis in order to increase the sample size as much as possible. Moreover, since the KOA also has male patients, we did not want to exclude male patients from the analysis in order to generalize the results of this study. However, as the reviewer pointed out, a sensitivity analysis was conducted to see if the results remained the same after excluding the males. We have revised the following sentences (please see **Page 14/Lines 290–292 in the Discussion section**).

“Moreover, women constitute a majority of the sample in this studied. Therefore, we conducted a sensitivity analysis to see if the results remained the same after excluding the males (Supplement Table 4).”

Moreover, the results are shown in Supplementary Table 4 (please see **Page 21/Lines 429–432 in the Supplemental data section**).

**Table 4.** Differences between women participants with mild and severe KOA in the quadriceps RFD and maximum quadriceps strength

|  | Mild KOA^*^ (n = 50) | Severe KOA^*^ (n=6) | p-value^†^ |
| --- | --- | --- | --- |
| Quadriceps RFD (%MVC/ms*kg) | 7.14±1.76 | 5.09±1.99 | **0.02** |
| Maximum quadriceps strength (Nm/kg) | 1.42±0.36 | 1.30±0.70 | 0.50 |

KOA: knee osteoarthritis; RFD: rate of force development

^*^ Values are expressed as mean ± SD or number (percentage)

^†^ Based on the unadjusted analysis (Mann–Whitney U-test) between participants with early and severe KOA

In terms of age, there was no significant difference in the results between mild and severe OA. However, previous studies have shown that the severity of KOA increases with age [1] and that muscle weakness becomes more pronounced with age [2]. For these reasons, we included age as a covariate.

**Reference**

1. Muraki S, Oka H, Akune T, Mabuchi A, En-yo Y, Yoshida M, et al. Prevalence of radiographic knee osteoarthritis and its association with knee pain in the elderly of Japanese population-based cohorts: the ROAD study. Osteoarthritis Cartilage. 2009;17:1137-43. doi: 10.1016/j.joca.2009.04.005.

2. Muraki S, Akune T, Teraguchi M, Kagotani R, Asai Y, Yoshida M, et al. Quadriceps muscle strength, radiographic knee osteoarthritis and knee pain: the ROAD study. BMC Musculoskelet Disord. 2015;16: 305. doi: 10.1186/s12891-015-0737-5.

**[Comment #21]**

Line 179: please put “effect size” before the abbreviation (ES)

**[Author action #21]**

Thank you for this important reminder. We have revised the following sentences (please see **Page 8/Lines 181–184 in the Materials and Methods section**).

“The effect size (ES), Hedges’ g, was calculated as the standardized mean difference (SMD) with mean ± SD and 95% CI using Review Manager Software (RevMan 5.3; Cochrane Collaboration, Oxford, UK) for the quadriceps RFD and maximum quadriceps strength.”

**[Comment #22]**

Line 180: please provide interpretation for Hedge’s g

**[Author action #22]**

Thank you for this important reminder. We have revised the following sentences (please see **Page 8/Lines 181–186 in the Materials and Methods section**).

“The effect size (ES), Hedges’ g, was calculated as the standardized mean difference (SMD) with mean ± SD and 95% CI using Review Manager Software (RevMan 5.3; Cochrane Collaboration, Oxford, UK) for the quadriceps RFD and maximum quadriceps strength. The Cohen scale was used to interpret ES, where 0.2 represents a small effect, 0.5 a moderate effect, and 0.8 a large effect.”

**[Comment #23]**

Table 1: for the sake of clarity add (%) in the “K & L grade” cell name

**[Author action #23]**

Thank you for this important reminder. We have revised the following sentences (please see **Page 9-10/Lines 207–212 in the Results section**).

**Table 1.** Characteristics of participants with mild and severe KOA

|  | Mild KOA^*^ (n = 58) | Severe KOA^*^ (n=8) | p-value^†^ |
| --- | --- | --- | --- |
| Age (years) | 74.05 ± 5.09 | 75.87 ± 4.45 | 0.23 |
| Women, n (%) | 50 (86.2) | 6 (75.0) | 0.59 |
| Height (cm) | 154.41±8.73 | 157.75±10.84 | 0.23 |
| Weight (kg) | 53.43±9.08 | 56.37±7.02 | 0.24 |
| BMI (kg/m^2^) | 22.35 ± 2.91 | 22.75 ± 2.72 | 0.62 |
| K&L grade, n (%) |  |  |  |
| Grade 1 | 17 (29.3) | − |  |
| Grade 2 | 41 (70.7) | − |  |
| Grade 3 | − | 6 (75.0) |  |
| Grade 4 | − | 2 (25.0) |  |
| Knee pain VAS (mm) | 25.06 ± 22.05 | 29.0 ± 23.13 | 0.56 |

BMI: body mass index; KOA: knee osteoarthritis; K&L grade: Kellgren and Lawrence grade; VAS: visual analog scale

^*^ Values are expressed as mean ± SD or number (percentage)

^†^ Based on the unadjusted analysis (Mann–Whitney U-test) of participants with early and severe KOA

**[Comment #24]**

Line 210-212: lower seems more appropriate than decreased since you were testing significance of differences. Also, something like “Participants with severe KOA had significantly lower quadriceps RFD (p=0.009) but not maximum strength (p=0.50) when compared to group with mild KOA (Table 2).” would read easier.

**[Author action #24]**

Thank you for this important reminder. We have revised the following sentences (please see **Page 10/Lines 215–216 in the Results section**).

“Participants with severe KOA had a significantly lower quadriceps RFD (p=0.009) but not maximum strength (p=0.50) when compared to group with mild KOA (Table 2).”

**[Comment #25]**

Table 2: unit for RFD doesn’t seem correct, please double-check

**[Author action #25]**

Thank you for this important reminder. We have revised the following sentences (please see **Page 11/Lines 227–230 in the Results section**).

**Table 2.** Differences between participants with mild and severe KOA in the quadriceps RFD and maximum quadriceps strength

|  | Mild KOA^*^ (n = 58) | Severe KOA^*^ (n=8) | p-value^†^ |
| --- | --- | --- | --- |
| Quadriceps RFD (%MVC/ms*kg) | 6.97±1.72 | 5.10±1.71 | **0.009** |
| Maximum quadriceps strength (Nm/kg) | 1.49±0.41 | 1.39±0.69 | 0.50 |

KOA: knee osteoarthritis; RFD: rate of force development

^*^ Values are expressed as mean ± SD or number (percentage)

^†^ Based on the unadjusted analysis (Mann–Whitney U-test) between participants with early and severe KOA

Moreover, in this study, RFD is corrected for maximum torque (%MVC) and body weight (kg). This correction was added to the Methods section. We have revised the following sentences (please see **Page 7/Lines 159–160 in the Materials and Methods section**).

“The RFD was normalized to body mass and MVC torque (%MVC) [20, 22].”

**[Comment #26]**

Table 2 shows MVC data normalized to body mass but such detail is missing from the methods section

**[Author action #26]**

Thank you for this important reminder. We have revised the following sentences (please see **Page 7/Lines 154–156 in the Materials and Methods section**).

“MVC in the quadriceps was defined as the maximal torque value obtained from two attempts. The MVC was normalized to body mass.”

**[Comment #27]**

Lines 227-235: Will all the respect to authors effort and work, this section is redundant it they should consider removing it. It does not provide any new information than the previous paragraph but only confirms the results of Man-Whitney test

**[Author action #27]**

Thank you for this important reminder. As explained in “Author action #20”, we believe that the relevant sections are necessary to generalize the results of this study. However, as the reviewer pointed out, this section is redundant. Therefore, we have made the following revisions.

Tables 3 and 4 have been moved to Supplemental Tables 1 and 2 (please see **Page 20/Lines 413–419 in the Supplemental data section**).

**Table 1.** Post hoc analysis to test the between-group differences adjusted for covariates in the quadriceps RFD for mild and severe KOA

|  | Mild KOA (n=58) | Severe KOA (n=8) | Between-group difference | 95% CI | Effect size | |
| --- | --- | --- | --- | --- | --- | --- |
|  |  |  |  |  | Hedges | 95% CI |
| Quadriceps RFD (%MVC/ms*kg) | 6.97±1.72 | 5.10±1.71 | **0.88** | **0.23–1.54** | **-1.07** | **-1.83 to -0.30** |

KOA: knee osteoarthritis; RFD: rate of force development; 95% CI: 95% confidence interval

Adjusted for age (years), sex, and knee pain VAS (mm)

**Table 2.** Post hoc analysis to test the between-group differences adjusted for covariates in maximum quadriceps strength for mild and severe KOA

|  | Mild KOA (n=58) | Severe KOA (n=8) | Between-group difference | 95% CI | Effect size | |
| --- | --- | --- | --- | --- | --- | --- |
|  |  |  |  |  | Hedges | 95% CI |
| Maximum quadriceps strength (Nm/kg) | 1.49±0.41 | 1.39±0.69 | 0.06 | -0.09 to 0.21 | -0.22 | -0.96 to 0.52 |

KOA: knee osteoarthritis; 95% CI: 95% confidence interval

Adjusted for age (years), sex, and knee pain VAS (mm)

Moreover, the results in this section have been modified to be presented in the same paragraph as the Mann-Whitney results. We have revised the following sentences (please see **Page 10/Lines 217–223 in the Materials and Methods section**).

“When adjusted for age, sex, and knee pain VAS, participants with severe KOA exhibited a significantly decreased quadriceps RFD (between-group difference: 0.88; 95% CI: 0.23 to 1.54). The quadriceps RFD exhibited a large effect (ES: -1.07; 95% CI: -1.83 to -0.30) (Supplemental Table 1). When adjusted for age, sex, and knee pain VAS, participants with severe KOA did not exhibit significantly decreased maximum quadriceps strength (between-group difference: 0.06; 95% CI: -0.09 to 0.21). Maximum quadriceps strength exerted a small effect (ES: -0.22; 95% CI: -0.96 to 0.52) (Supplemental Table 2).”

**[Comment #28]**

Lines 260-262: not decrease but difference. This study shows no difference between mild and severe KOA in maximum strength, but significant difference in RFD.

**[Author action #28]**

Thank you for this important reminder. We have revised the following sentences (please see **Page 12/Lines 240–242 in the Discussion section**).

“Moreover, the difference in the quadriceps RFD was greater than the difference in maximum quadriceps strength in individuals with severe KOA based on a comparison of the ES between the quadriceps RFD and maximum quadriceps strength.”

**[Comment #29]**

Line 281: Such statement would make sense if this was longitudinal study or if you had age/gender/physical activity etc. -matched controls. Maybe, just maybe, the maximum strength deteriorates to its lowest at stage of mild KOA and only RFD keeps deteriorating from there on (please refer to lines 270-271 where you discuss the influence of fiber type)

**[Author action #29]**

Thank you for this important reminder. We have revised the following sentences (please see **Page 12/Lines 261–262 in the Discussion section**).

“In this study, participants with severe KOA did not exhibit significantly differences in the maximum quadriceps strength.”

**[Comment #30]**

Lines 285-287 only repeat what was already said in lines 281-282

**[Author action #30]**

Thank you for this important reminder. The relevant sentence has been deleted from the manuscript.

**[Comment #31]**

Lines 288-290: The positioning of such statement is not clear. It only repeats what was already stated above.

**[Author action #31]**

Thank you for this important reminder. The relevant sentence has been deleted from the manuscript.
